# Spatial and temporal patterns of dengue incidence in northeastern Thailand 2006–2016

**DOI:** 10.1186/s12879-019-4379-3

**Published:** 2019-08-23

**Authors:** Thipruethai Phanitchat, Bingxin Zhao, Ubydul Haque, Chamsai Pientong, Tipaya Ekalaksananan, Sirinart Aromseree, Kesorn Thaewnongiew, Benedicte Fustec, Michael J. Bangs, Neal Alexander, Hans J. Overgaard

**Affiliations:** 1 Department of Microbiology, Khon Kaen University, Khon Kaen, Thailand; 2 Department of Medical Entomology, Faculty of Tropical Medicine, Mahidol University, Bangkok, Thailand; 3 Department of Biostatistics, University of North Carolina at Chapel Hill, Chapel Hill, USA; 4 Department of Biostatistics and Epidemiology, University of North Texas Health Science Center, Fort Worth, TX USA; 5 HPV & EBV and Carcinogenesis Research Group, Khon Kaen University, Khon Kaen, Thailand; 6 Department of Disease Control, Office of Disease Prevention and Control, Region 7 Khon Kaen, Ministry of Public Health, Khon Kaen, Thailand; 7 Université de Montpellier, Montpellier, France; 8 Public Health & Malaria Control, PT Freeport Indonesia, International SOS, Kuala Kencana, Papua Indonesia; 9 London School of Hygiene and Tropical Medicine, London, UK; 10 Faculty of Science and Technology, Norwegian University of Life Sciences, Ås, Norway

**Keywords:** Dengue, Climate, Seasonal, Temperature, Rainfall, Thailand

## Abstract

**Background:**

Dengue, a viral disease transmitted by *Aedes* mosquitoes, is an important public health concern throughout Thailand. Climate variables are potential predictors of dengue transmission. Associations between climate variables and dengue have usually been performed on large-scale first-level national administrative divisions, i.e. provinces. Here we analyze data on a finer spatial resolution in one province, which is often more relevant for effective disease control design. The objective of this study was to investigate the effect of seasonal variations, monthly climate variability, and to identify local clusters of symptomatic disease at the sub-district level based on reported dengue cases.

**Methods:**

Data on dengue cases were retrieved from the national communicable disease surveillance system in Thailand. Between 2006 and 2016, 15,167 cases were recorded in 199 sub-districts of Khon Kaen Province, northeastern Thailand. Descriptive analyses included demographic characteristics and temporal patterns of disease and climate variables. The association between monthly disease incidence and climate variations was analyzed at the sub-district level using Bayesian Poisson spatial regression. A hotspot analysis was used to assess the spatial patterns (clustered/dispersed/random) of dengue incidence.

**Results:**

Dengue was predominant in the 5–14 year-old age group (51.1%). However, over time, dengue incidence in the older age groups (> 15 years) gradually increased and was the most affected group in 2013. Dengue outbreaks coincide with the rainy season. In the spatial regression model, maximum temperature was associated with higher incidence. The hotspot analysis showed clustering of cases around the urbanized area of Khon Kaen city and in rural areas in the southwestern portion of the province.

**Conclusions:**

There was an increase in the number of reported dengue cases in older age groups over the study period. Dengue incidence was highly seasonal and positively associated with maximum ambient temperature. However, climatic variables did not explain all the spatial variation of dengue in the province. Further analyses are needed to clarify the detailed effects of urbanization and other potential environmental risk factors. These results provide useful information for ongoing prediction modeling and developing of dengue early warning systems to guide vector control operations.

**Electronic supplementary material:**

The online version of this article (10.1186/s12879-019-4379-3) contains supplementary material, which is available to authorized users.

## Background

The annual global burden of dengue is estimated at 390 million infections, of which 96 million present clinically [1]. Four closely related RNA viruses in the family *Flaviviridae* (DENV1 to DENV4) are responsible for dengue disease. They are transmitted by *Aedes* (primarily subgenus *Stegomyia*) mosquitoes, particularly *Aedes aegypti* (L.) and *Aedes albopictus* (Skuse) [2]. Dengue has developed from a sporadically occurring disease to a major and re-emerging global public health problem over recent decades causing substantial economic disruption and social burden in endemic areas in Asia, Africa, and the Americas. There is no effective treatment for dengue and vaccination, so far, offers only incomplete protection [3, 4]. Therefore, vector control remains the most important means of prevention [5]. Effective vaccine or not, vector control will remain the cornerstone of dengue control for years to come [3].

Due to increasing incidence and rapid geographical expansion, dengue is the most common vector-borne disease in Thailand [6]. From 2000 to 2011, the number of reported cases varied from 20,000 to 140,000 cases each year [7]. Both *Ae. aegypti* and *Ae. albopictus* are common species and widely distributed in Thailand [8]. All four serotypes co-circulated in each of the major outbreaks that occurred in 1958, 1987, 1998, 2001, 2013, and 2015 [9–14]. The highest incidence typically occurs in 13–24 year-old age group with case clustering seen predominately in urban areas [15]. Males represent the majority of reported dengue cases in several Asian countries [16]. A study in Singapore showed that men were more exposed to infected mosquitoes than women, during daytime hours, at the workplace or while travelling to and from work. A forceful public health policy in Singapore [17] has greatly reduced the number of mosquitoes in and around homes, potentially rendering the larger male labor force more exposed to mosquito bites during working hours [16, 18]. Other causes for these apparent gender differences could be different health seeking behaviors or male-female differences in disease severity [19]. In the Lao People’s Democratic Republic male-female ratios in dengue cases varied between years and provinces [16]. We are not aware of similar spatio-temporal or socioeconomic differences in Thailand.

Thailand has adapted the dengue control strategy of the World Health Organization (WHO) [2], which consists of three main pillars: 1) patients diagnosed with dengue are required to avoid mosquito bites to prevent dengue transmission; 2) active community case detection of cases which do not result in clinical consultation; and 3) vector control, consisting of environmental management, source reduction, and chemical interventions using insecticide fogging against adult vectors and larvicides to control immature stages in containers [20]. Follow-up interventions are conducted by health officers or village health volunteers [20]. To determine the most appropriate and feasible intervention or combination of interventions, health officers need to consider local environmental, resource, and contextual factors that may influence effectiveness [21].

Climate variables are predictors of dengue infection [4, 22, 23]. Seasonal variation in climate shows a strong relationship with *Ae. aegypti* abundance and historical dengue incidence [24]. Temperature affects population biology of *Aedes* mosquitoes [25]. Higher temperatures increase larval development [26] and rates of multiple feeding, but reduce mosquito size [27]. The extrinsic incubation period declines as temperature rises, thus increasing the proportion of infected vectors, and enhancing the transmission potential of the vector [27–29].

As ambient temperature increases, so does dengue epidemic potential, peaking at around 29 °C and then decreases [29]. In subtropical and tropical regions such as Thailand, with mean diel temperatures of 26 °C (20 °C ≤ T ≤ 32 °C), an increase in diurnal temperature range can enhance transmission [29]. An analysis of data from Thailand (1978–1997) showed the incidence of dengue hemorrhagic fever (DHF) was negatively associated with higher rainfall in the southern region of the country, but positively associated with elevated ambient temperatures in the central and northern regions [30]. Another study using provincial monthly dengue data from 1983 to 2001 concluded that the relationships between weather variables and dengue transmission are very complex in Thailand [31]. The study found that transmission occurs within a specific temperature range, but that changes in humidity within this range can amplify the transmission potential with 80% of dengue cases occurring at a mean temperature of between 27.0 and 29.5 °C and a mean relative humidity of > 75%. They further found that large epidemics begin earlier, develop faster and can be predicted at a defined onset time. Non-linear modeling of more than 30 years (1982–2013) of monthly data by province in Thailand showed that inter-annual variations in rainfall and temperature with a lag time of one month can improve the explanation of dengue relative risk compared to a seasonal-spatial model [32]. The relationship between rainfall and dengue is complex, as it may create abundant breeding sites for the vector [33], but can also flush out sites if rain is too intense [33, 34]. Because household water storage may increase in the dry season, the resulting breeding habitats may weaken, or even reverse, the positive association between dengue and rainfall [35–39].

Spatio-temporal analysis can detect clusters of dengue disease and is useful for a better understanding of the dynamics of disease dispersion. Analysis of spatial and temporal variations is also useful in identifying high-risk locations and times of higher transmission risk, which are important for disease surveillance and control [15, 40].

The above-mentioned research on climate and dengue focused on larger spatiotemporal scales, such as monthly dengue surveillance and climate records at the provincial level [31, 41, 42]. The current study is novel because it uses data on the lowest administrative level, the sub-district, in one province to understand fine-scale spatial dengue-climate relationships. This is useful for developing more reliable prediction models for future projections applied in early warning and response systems, thus ultimately improving timely control interventions.

We analyzed data on reported dengue cases in Khon Kaen Province, northeastern Thailand collected between 2006 and 2016 to 1) describe demographic characteristics and seasonal variations of dengue cases; 2) determine the potential impact of climate variability on dengue incidence; and 3) identify clusters of dengue cases at the sub-district level.

## Methods

### Study area

The study was conducted in Khon Kaen Province, an area of approximately 10,900 km^2^ (16°25′12″N to 16°42′12″N and 102°49′48″E to 102°83′48″E). The province has 26 districts, 199 sub-districts, and 2139 villages. In 2010, the population was 1,767,601, of which 387,279 people lived in Mueang District that includes the provincial capital Khon Kaen (see Additional file 1). This province was selected as the study area because dengue is endemic with typical seasonal increases and occasional outbreaks. The province is primarily rural with a few large urban centers. Mueang District, the most densely populated area in the province, is a regional center for education, health, finance and commerce. The northern and southern parts of the district, along the major highway linking Bangkok with Lao People’s Democratic Republic, are rapidly developing. The districts in the northwestern and southeastern parts of the province are rural and agricultural. Classification of urban and rural areas depends on population density. An urban area is defined as a municipality or town with a population over 100,000 and a population density above 300 persons per square kilometer [43]. The average minimum and maximum seasonal temperatures are 16.7 °C (December–January) and 36.4 °C (April–May). The monthly minimum and maximum rainfall vary from 0 mm (dry season: November–April) to 240 mm (wet season: May–October).

### Data collection

The Office of Disease Prevention and Control, Region 7 Khon Kaen (ODPC7), Department of Disease Control, Ministry of Public Health, Thailand provided data on the weekly number of reported dengue cases in Khon Kaen Province from 1 January 2006 to 31 December 2016. Dengue is a notifiable disease based on the National Communicable Disease Control Law, i.e., all government and private hospitals, clinics and other healthcare facilities must report all cases (confirmed and suspected) to the local health authority within 24 h of diagnosis [12]. Cases are recorded by degree of disease severity into one of three categories (at peak of illness): 1) dengue fever (DF), 2) dengue hemorrhagic fever (DHF), and 3) dengue shock syndrome (DSS), but the serotype is not recorded (or typically known except retrospectively). A patient is diagnosed with suspected DF when the following criteria are met and signs and symptoms are present: residence or recent travel to a dengue endemic area, acute fever accompanied by any two of the following: headache, myalgia, arthralgia, rash, positive tourniquet test and leucopenia, with no evidence of plasma leakage. DHF is recorded in patients with a temperature ≥ 38 °C, petechiae, ecchymosis, or a positive tourniquet test, thrombocytopenia (platelets < 100,000 cells/mm^3^), and evidence of plasma leakage. DSS, the most severe disease manifestation, is defined as having the same signs and symptoms as DHF, but progressing to circulatory failure. The Provincial Health Offices enter patient data into the standardized Disease Surveillance Report (Report 506) for recording communicable diseases in Thailand. The form provides the patient’s age, gender, house address, signs and symptoms, and date of medical consultation. DHF and DSS are based on both clinical symptoms and laboratory tests (usually complete blood count), and sometimes accompanied with a rapid diagnostic test (RDT); whereas, DF is seldom based on additional laboratory tests or by RDT.

Meteorological data from 1 January 2006 to 31 December 2016 were downloaded from the data library of the International Research Institute for Climate and Society [44], which contains specific climate data from different sources, such as The National Centers for Environmental Prediction (NCEP), Climate Forecast System Reanalysis (CFSR) [45], and Climate Hazards Group InfraRed Precipitation with Station data (CHIRPS) global rainfall datasets [46]. For each sub-district, daily temperatures (°C) were retrieved from NCEP and daily rainfall (mm) from CHIRPS. These data generated the monthly means used in the analysis (see Additional files 2 and 3). The spatial resolution of rainfall is 0.05 × 0.05 degrees (CHIRPS) and for temperature 0.2 × 0.2 degrees (NCEP CFSR v2, https://rda.ucar.edu/datasets/ds094.1/). A centroid was created for each sub-district. Rainfall and temperature data for each sub-district was determined based on the grid cell in which the centroid was located.

### Analysis

Monthly data on dengue cases and climate (rainfall and temperature) from the study period were combined to visualize seasonal patterns and temporal trends. Dengue incidence was calculated using the monthly number of reported cases and sub-district population size in 2010 reflecting the mid-study denominator [47].

Bayesian Poisson regression models were used to assess associations with the number of monthly cases in 199 sub-districts. Population was used as the denominator in the model (i.e. log-population as an offset). The neighborhood relationship between the sub-districts were defined using their adjacency matrix; ‘1’ for a pair of sub-districts sharing a border, otherwise ‘0’. Hence the following model was used:

$$ {Y}_{ij}\sim Poisson\ \left({\mu}_{ij}\right) $$

$$ \log\ \left({\mu}_{ij}\right)=\log\ \left({P}_i\right)+{\theta}_{ij} $$

$$ {\theta}_{ij}=\alpha +{\beta}_k{x}_{ij k}+{u}_{ij}, $$

where *Y*_*ij*_ is the observed mean number of cases for the *i*th sub-district in *j*th month (*i* = 1, …,199; *j* = 1, …,12), *P*_*i*_ is the sub-district population size, *α* is the intercept, and *β*_*k*_ is the regression coefficient for covariate *k*. For the main model, the covariates (*x*_*k*_) were: population density per square kilometer; gender (proportion of males among the cases), mean age in years of the cases; mean rainfall; and minimum and maximum temperature. As a non-mechanistic way of measuring the seasonality of incidence, a second set of covariates was obtained by replacing three meteorological variables by sine and cosine terms with period 12 months. Finally, *u*_*ij*_ is the random effect that captures the spatio-temporal autocorrelation in response data *Y*_*ij*_, whose variance depends on the adjacency matrix.

Conditional autoregressive (CAR) priors [48] structure were used on *u*_*ij*_ and for (*α*, *β*_*k*_), non-informative normal prior distributions was used. Flat and conjugate priors were specified for *u*_*ij*_ using inverse gamma distributions with shape and scale parameters equal to 0.001. Markov chain Monte Carlo simulation was used to estimate the model parameters, sampling 300,000 times, with the first 150,000 as the burn-in, and keeping the results from every tenth iteration. The “ST.CARar” function of the R statistical software package CARBayesST (www.r-project.org) was used to fit the model. Convergence was assessed by trace plots and checked by the convergence Z-score diagnostic function [49]. The Watanabe-Akaike Information Criterion (WAIC) was used as a measure of goodness of fit [50].

Local Indicators of Spatial Association (LISA) were used to identify significant hotspots, coldspots, and outliers of dengue incidence at the sub-district level [51]. A hotspot is defined as an area that is surrounded by other high incidence areas, i.e. incidence is higher than the expected number given a random distribution of cases (so called high-high cluster). A coldspot is defined as an area surrounded by other low incidence areas (low-low cluster). Hotspot detection can be useful, even if the global pattern is not clustered. Moreover, case clusters that occur randomly can also have an influence on the spread of an infectious disease [52].

## Results

### General results

**Fig. 1:**
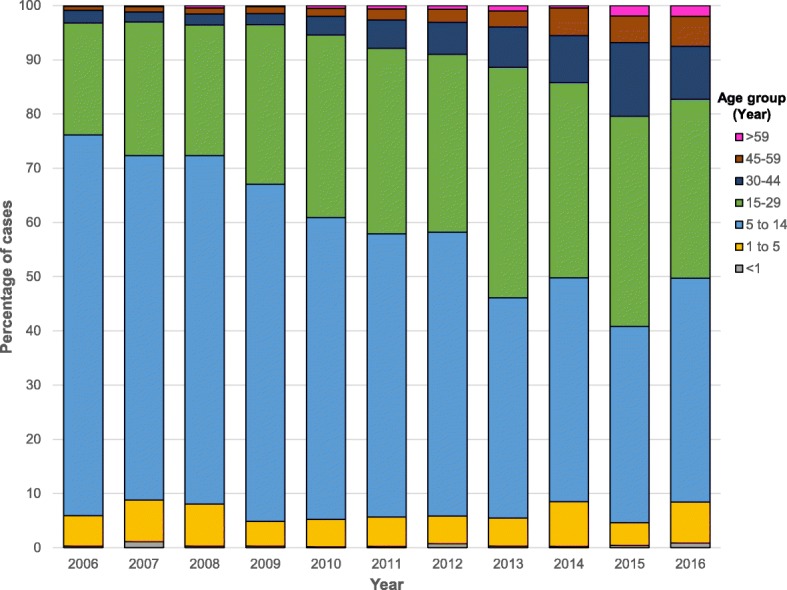
Age distribution of reported dengue cases (DF, DHF and DSS) in Khon Kaen Province, Thailand, 2006–2016

**Fig. 2:**
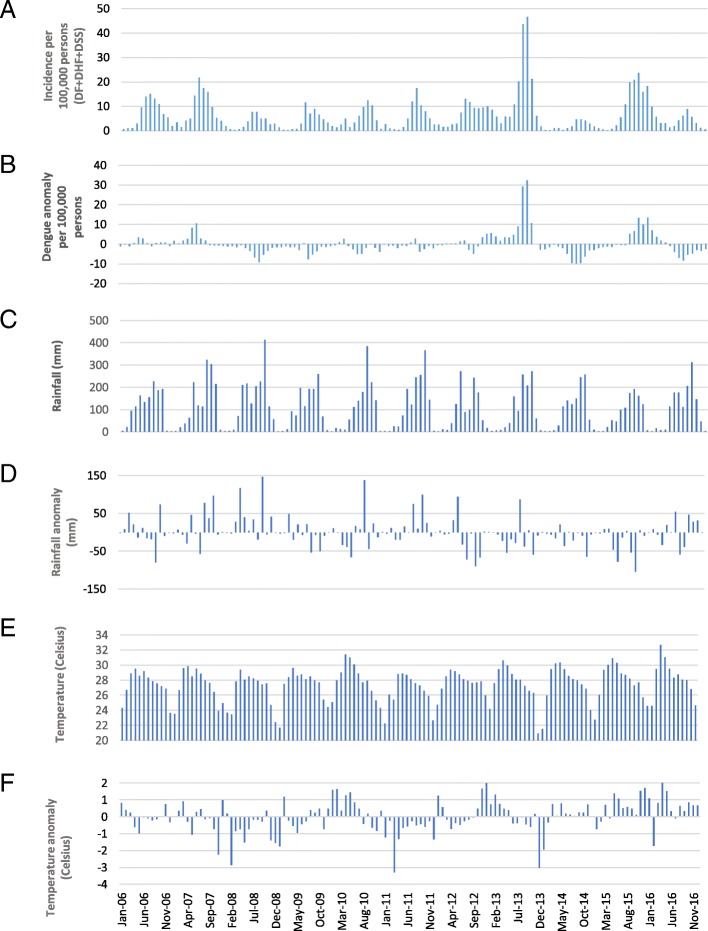
Monthly dengue incidence (**a**), dengue anomaly (**b**), rainfall (**c**), rainfall anomaly (**d**), temperature (**e**) and temperature anomaly (**f**) in Khon Kaen Province, Thailand, 2006–2016. DF = dengue fever, DHF = dengue hemorrhagic fever, DSS = dengue shock syndrome

**Fig. 3:**
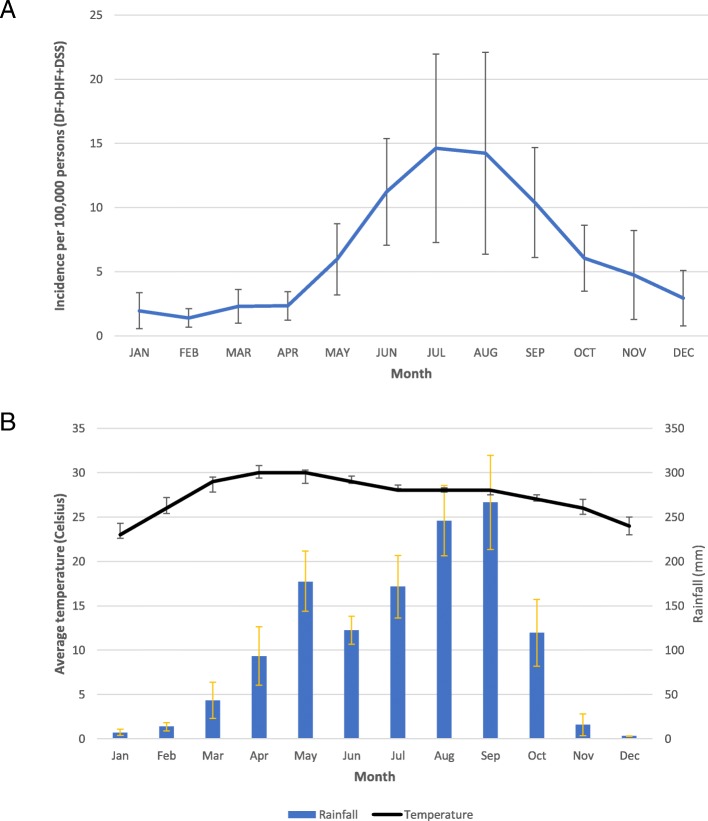
Mean monthly dengue incidence per 100,000 persons (**a**) and monthly average of rainfall (bar) and temperature (line) (**b**) in Khon Kaen Province, Thailand, 2006–2016

Dengue cases numbering 15,167 were reported over the 11-year period by all hospitals and clinics in Khon Kaen Province. Of these, there were 7461 dengue fever cases (49.2%) and 7706 severe dengue cases (50.8%), comprising both DHF and DSS. The demographic characteristics of patients are summarized in Table 1. Males represented the majority of patients (8057; 53.1%). Ages ranged from 4 months to 92 years old (median 13 years). The highest number of patients was in the 5–14 year-old age group (7758; 51.1%), followed by 15–29 years (5026; 33.1%) and 30–44 years (937; 6.2%). The proportion of older age groups (> 15 years), increased from nearly 20% of all cases in 2006 to more than 50% in 2016 (Fig. 1). The highest recorded disease incidence was in 2013, approximately 80 per 100,000 population (Fig. 2). Incidence was high during the rainy season (May–September), with July having the highest incidence (Fig. 3).

**Table 1:** Demographic characteristics of dengue reported cases in Khon Kaen Province, Thailand, 2006–2016

Characteristics	Number of cases	Percentage (%)
Gender		
Male	8057	53.1
Female	7110	46.9
Age group (years)		
< 1	72	0.5
1–<5	857	5.7
5–<15	7758	51.1
15–<30	5026	33.1
30–<45	937	6.2
45–<60	391	2.6
> 60	126	0.8
Diagnosis		
Dengue fever (DF)	7461	49.2
Dengue haemorrhagic fever (DHF)	7186	47.4
Dengue shock syndrome (DSS)	520	3.4

### Association between dengue cases and climatic factors

Mean rainfall and maximum temperature were positively associated with dengue incidence, and minimum temperature was negatively associated, in terms of their point estimates (Table 2). However, among the three 95% credible intervals (CIs), only the one for maximum temperature excluded 1 (null effect). The rate ratio for maximum temperature was 1.055, implying 5.5% (95% CI 0.9–11.5%) increase in cases with an increase of 1 °C per month. The range of this variable was from 30.7 °C to 44.9 °C. The rate ratio for mean rainfall was 1.004, indicating that increasing rainfall by one unit (1 cm) per month would increase dengue incidence by about 0.4%. The Watanabe-Akaike Information Criterion (WAIC) for this model was 10,028.75. For the model with two sinusoid terms replacing the three meteorological variables, the WAIC was very similar, at 10028.23. This sinusoid terms had a peak to trough rate ratio of 5.8, and a peak in mid-July, i.e. a roughly six-fold difference in fitted incidence from mid-July to mid-January.

**Table 2:** Point estimates and 95% credible interval of the Bayesian Poisson regression model on number of all monthly dengue cases (DF, DHF and DSS) and covariates in Khon Kaen Province, Thailand, 2006–2016

Parameter	Rate ratios		
	Median	2.5%	97.5%
Mean monthly rainfall (cm)	1.004	0.990	1.017
Maximum temperature (°C)	1.055	1.009	1.115
Minimum temperature (°C)	0.958	0.927	1.024
Age (years)	0.990	0.985	0.994
Gender (proportion female^a^)	0.933	0.854	1.020
Density (thousands of people per km^2^)	0.925	0.827	1.047

^a^Hence the rate ratio is for 100% female case composition relative to 100% male case composition

**Fig. 4:**
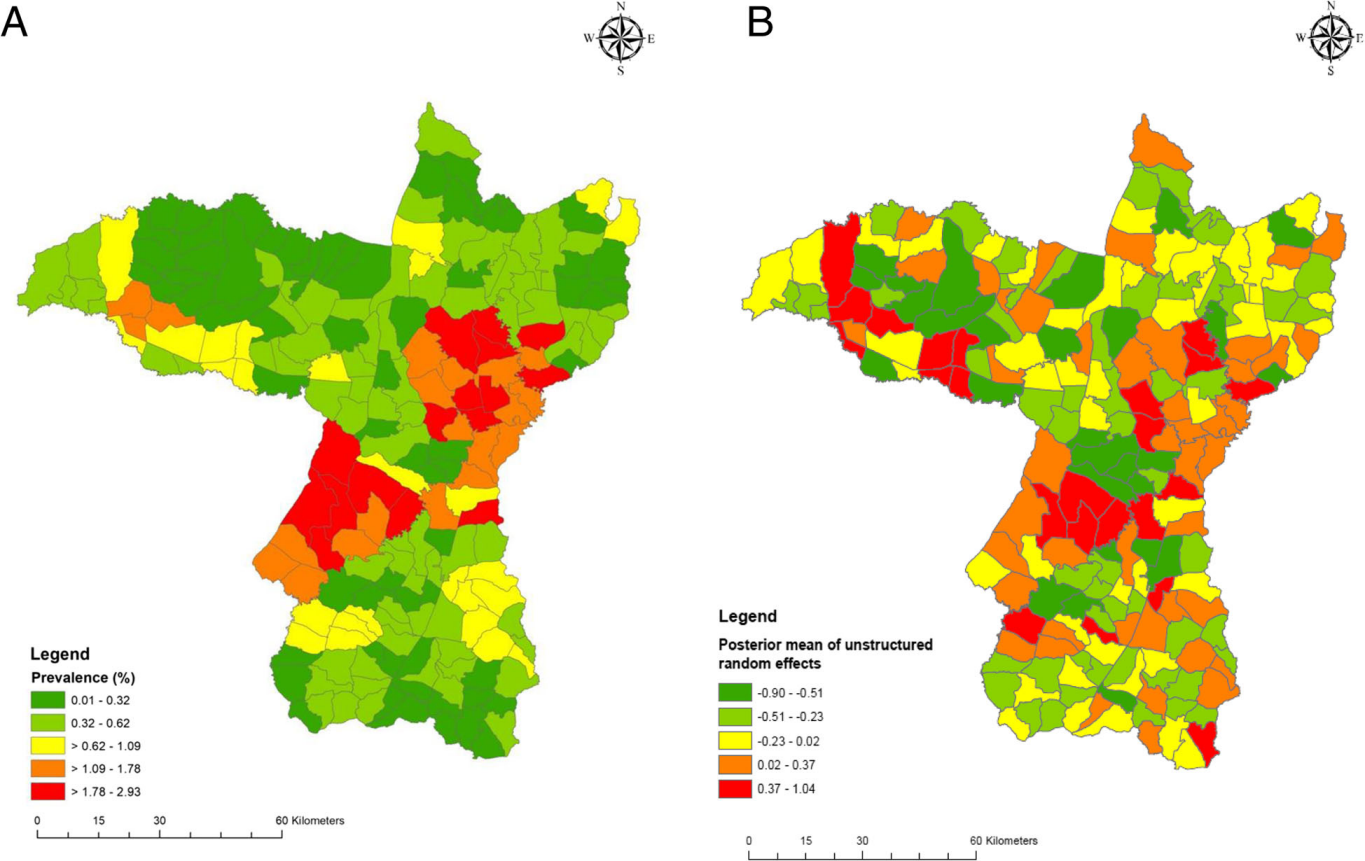
Mean dengue prevalence by sub-district (**a**) and spatial distribution of the posterior means of random effects for dengue (**b**) in Khon Kaen Province, Thailand, 2006–2016

**Fig. 5:**
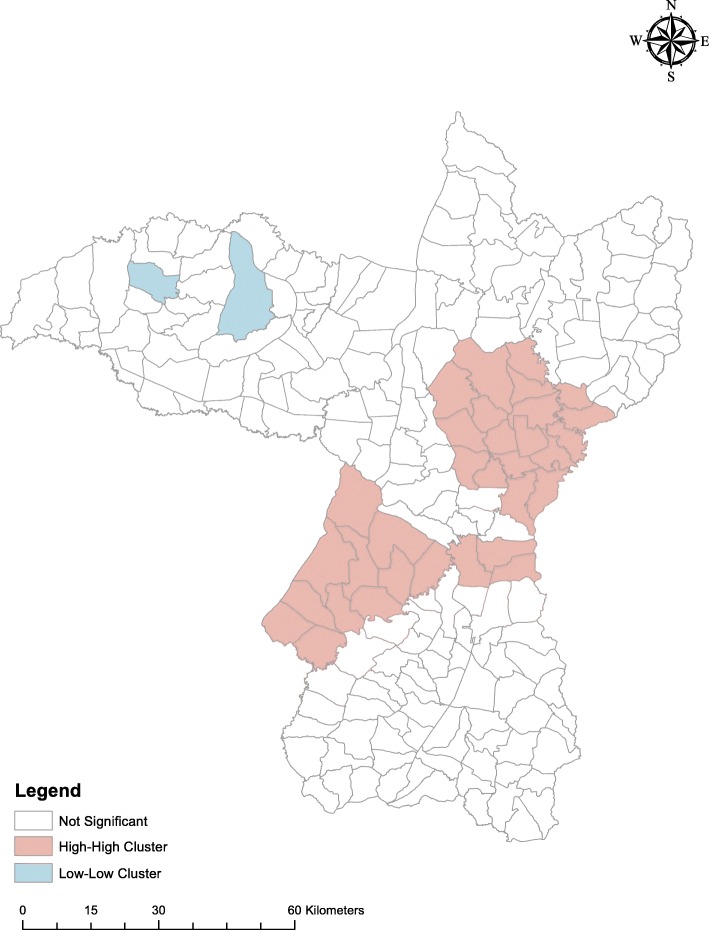
High and low clustering of dengue incidence in Khon Kaen Province, Thailand, 2006–2016

The mean dengue incidence was high in the central northeastern sub-districts, around Khon Kaen city, and in the southwestern sub-districts of the province (red and orange in Fig. 4a). The distribution of the posterior means of the random effects (from the CAR model with meteorological variables) show some clustering, indicating that the variables in the model did not account fully for the spatial variation in the data (Fig. 4b). Posterior distribution plots are shown in Additional file 4. High clusters were present around Khon Kaen city and the southwestern portion of the province and low clusters were present in the northwestern area (Fig. 5), from the LISA analysis. When broken down by month, the incidences show the same clustering patterns, especially during July–August (Additional file 5).

## Discussion

The majority (~ 90%) of patients were below the age of 30 years. The trend during the study period showed that the proportion of dengue cases younger than 15 years declined from almost 80% in 2006 to below 50% in 2016. Dengue fever is generally more common in younger age groups [53], although there is evidence showing increasing incidence of more severe disease and outcomes among older age groups [54]. Our observations are also consistent with a population age shift, potentially influenced by demographic changes, such as the birth and death rates that show decreasing trends during 2011 and 2015 [55]. Thailand, in general, is undergoing a demographic transition where the proportion older adults are gradually increasing with an increase in median age of the general population. A higher proportion of adults will also increase the number of immune individuals (those with previous exposure to dengue virus) in the population, which might theoretically decrease the risk of dengue infection in younger people by providing alternative blood sources for infectious mosquitoes [56]. This age shift has also been observed in other Asian countries with a higher frequency of dengue cases among people 15 years of age and older [16]. Increases in disease incidence in older age groups may be explained by an increase in secondary infections and changes in circulating dengue virus serotypes [57], which have been shown to be important risk factors for severe clinical presentations [58–62].

There were clear seasonal patterns of dengue incidence in Khon Kaen Province during the study period. Dengue occurs throughout the rainy season, with 73% of cases reported between May and September. Although maximum temperature was associated with higher incidence (Table 2), the model with meteorological covariates had similar performance (in terms of the WAIC) to a non-mechanistic model, which simply fitted a sinusoidal pattern with a period of 12 months. In our study, a 1 cm increase in monthly rainfall was associated with a 0.4% increase in dengue incidence. In Timor Leste, results from similar modeling analyses showed a far larger effect: a 47% increase in incidence per 1 mm increase in annual rainfall [63]. Different climate patterns between Timor Leste and Thailand might explain these differences. Rainfall can affect the availability of mosquito larval habitats [34]. During rainy and dry periods of the year, permanent water containers are common in and around households; some located in toilet or bathroom spaces providing continuous year round mosquito production [35–39, 64]. Large water storage jars and tanks are the most commonly used containers in Thailand [64]. A study correlating rainfall and clinical dengue cases in Thailand from 2002 to 2003 also found that the dengue incidence was closely related with rainfall [65].

Temperature is another primary environmental risk factor for dengue transmission. Sea surface temperature (SST) changes, generally related to periodic El Niño Southern Oscillation effects, and air temperature, having more direct short-term effects, have both been shown to influence dengue incidence [63, 66]. Dengue incidence increased by 19.4% with a 1 °C increase in SST and 2.6% with a 1 °C increase in weekly maximum temperature in the Texas-Mexico border region [66]. Another study found that a 1 °C monthly increase in mean ambient temperature, dengue incidence increased by 0.7% [63]. In our study, the rate ratio for maximum temperature was 1.055 per °C, within the range from 30.7 °C to 44.9 °C. Higher temperatures enhance viral replication in the vector mosquito in a shorter amount of time and thus increase transmission potential of dengue viruses. A study of the extrinsic incubation period (EIP) of dengue serotype 2 in *Aedes albopictus* found that the virus remained in the midgut at 18 °C but could disseminate and invade the salivary glands at temperatures between 23 °C and 32 °C [67], thereby showing higher temperatures produce a shorter EIP and greater transmission potential. The strong and consistent relationships between climate, particularly rainfall and temperature, and the number of dengue cases have been used to develop prediction models to implement more timely dengue control measures [68, 69]. Relationships between dengue transmission and climatic variables have been examined in numerous studies, as shown above, but the question remains how to use such relationships in predicting impending outbreaks and applying effective interventions in time to avert them. User-friendly tools, such as the operational guide on Early Warning and Response System developed with support from the WHO/TDR and the European Union [70], are needed and will be tested in forthcoming work in Khon Kaen Province.

The highest dengue incidence seen in this study occurred in two areas of the province: around Khon Kaen Mueang District in the northeast, and in Manchakhiri and Khokphochai districts in the southwest. Mueang District includes the provincial capital and has the highest human population density, and in general, more conducive to dengue transmission. Manchakhiri and Khokphochai districts have lower population densities, but are, from our observations, seemingly similar to other districts in the province, i.e. vector species are present, larval habitats are plentiful, with a susceptible human population; therefore there must be other yet unexplored factors that support high dengue transmission in these two districts.

Although dengue incidence is influenced by rainfall and temperature, in our data there is no apparent spatial clustering of cases associated with the spatial variability in these environmental parameters. Rather, other factors such as urbanization are likely causes of the observed clustering effect [71]. However, population density, which was included in the regression model as a measure of urbanization, was not independently associated with dengue incidence. The residual spatial variation visible in Fig. 4b suggests that variables beyond those included in the spatial regression model are needed to explain differences in incidence between urban and rural subdistricts. Moreover, hotspots in more rural areas of southwestern Khon Kaen Province, further corroborate the influence of factors other than urbanization driving transmission. We do not know of any specific reasons for why these rural areas should have elevated dengue prevalence. One speculation could be that the lakes and swamps that are common in this area may provide suitable humidity for mosquitoes to thrive, but this was not studied here. Large changes in population size over time will affect outcomes. However, during 2000 and 2015, the average annual population growth rate in Thailand was less than 0.5% [72], which might not have affected the results substantially. Rural-urban migration is common in Thailand, with people drawn by, for example, better education, job opportunities, health facilities, standard of living, and wages [73]. Human movement is also an important factor in the dynamics of dengue transmission [74]. Adults are more likely to have greater mobility than younger age groups; therefore, to understand the circulation of the virus information on recent travel history and working conditions (location, time of work, etc.) is required. Elsewhere in Thailand, greater vulnerability to dengue infection has been observed in villages situated closer to urban centers [75]. Such neighboring effects are related to similarities in human behavior, development infrastructure, and ecological surroundings. Moreover, similar lifestyles and social interactions between neighboring areas are evident between villages that share social and religious centers such as schools, temples, mosques and community halls [75]. Hence, the results presented here are generalizable to most of northern Thailand, Laos, and Cambodia, and potentially Vietnam and Myanmar as well, under similar epidemiological settings.

Data collected from national surveillance systems come with inherent limitations, including underreporting and misreporting of symptomatic cases as well as the absence of subclinical and asymptomatic infections [76]. Moreover, dengue cases are seldom laboratory confirmed or identified to serotype. Another limitation of this study is inaccuracy, albeit minor, of the population denominators within sub-districts, as these were taken as fixed values from a single census (2010). Lastly, the possibility of travel-related infections was not determined in this study, which would provide potential misclassification bias. Nationally, the importance of travel-related dengue would vary by locality based on mobility. Obviously, we cannot exclude the possibility that some dengue infections were acquired outside the study area, thus potentially affecting the analysis and conclusions. However, if the general travel patterns had not changed significantly over the 11-year observation period, the dengue disease trends reported in this study would remain valid.

## Conclusion

We examined the epidemiology of dengue in Khon Kaen Province, Thailand between 2006 and 2016. There was an increase in older age groups reporting symptomatic dengue. Symptomatic dengue disease in people > 15 years of age is now more common than in children in this province, an observation that has been seen in other Asian countries. This study used monthly sub-district level data to show that rainfall and temperature have significant effects on dengue transmission in the province. Spatial clustering of cases is partly associated with urban areas closer to Khon Kaen city and rural areas in the southwest of the province. However, the current analysis was not able to detect a close proxy factor to quantify a relationship between urbanization and dengue incidence. The data set awaits further analysis for temporal patterns of infection for use in disease prediction modeling and developing dengue early warning systems to guide vector control operations.

## Additional files

Additional file 1: Population density per sub-district, Khon Kaen province, Thailand, 2006 to 2016. Location of province in northeastern Thailand (inset). (PDF 128 kb)

Additional file 2: Average monthly temperature (°C) per sub-district, Khon Kaen province, Thailand, January to December 2006–2016. (PDF 51 kb)

Additional file 3: Average monthly rainfall (mm) per sub-district, Khon Kaen province, Thailand, January to December 2006–2016. (PDF 49 kb)

Additional file 4: Posterior distribution plots of A) Mean rainfall, B) Minimum temperature, C) Maximum temperature, D) Age, E) Gender, and F) Population density. (PDF 265 kb)

Additional file 5: Average monthly incidence of dengue (DF, DHF, and DSS) per 10,000 persons in Khon Kaen province, Thailand, January to December 2006–2016. (PDF 58 kb)

## Data Availability

The datasets used during the current study are available from the corresponding author on reasonable request.

## Abbreviations

CAR: Conditional autoregressive
DF: Dengue fever
DHF: Dengue hemorrhagic fever
DSS: Dengue shock syndrome
EIP: Extrinsic incubation period
ODPC7: Office of Disease Prevention and Control 7
SST: Sea surface temperature
