# Supplementary material for: Spatial and temporal patterns of dengue incidence in northeastern Thailand 2006–2016
Source: BMC Infect Dis. 2019 Aug 23;19:743. doi: 10.1186/s12879-019-4379-3 (PMC6708185; doi:10.1186/s12879-019-4379-3)
Supplement: Supplementary file 1 — Population density per sub-district, Khon Kaen province, Thailand, 2006 to 2016. Location of province in northeastern Thailand (inset). (PDF 128 kb) [file 12879_2019_4379_MOESM1_ESM.pdf]

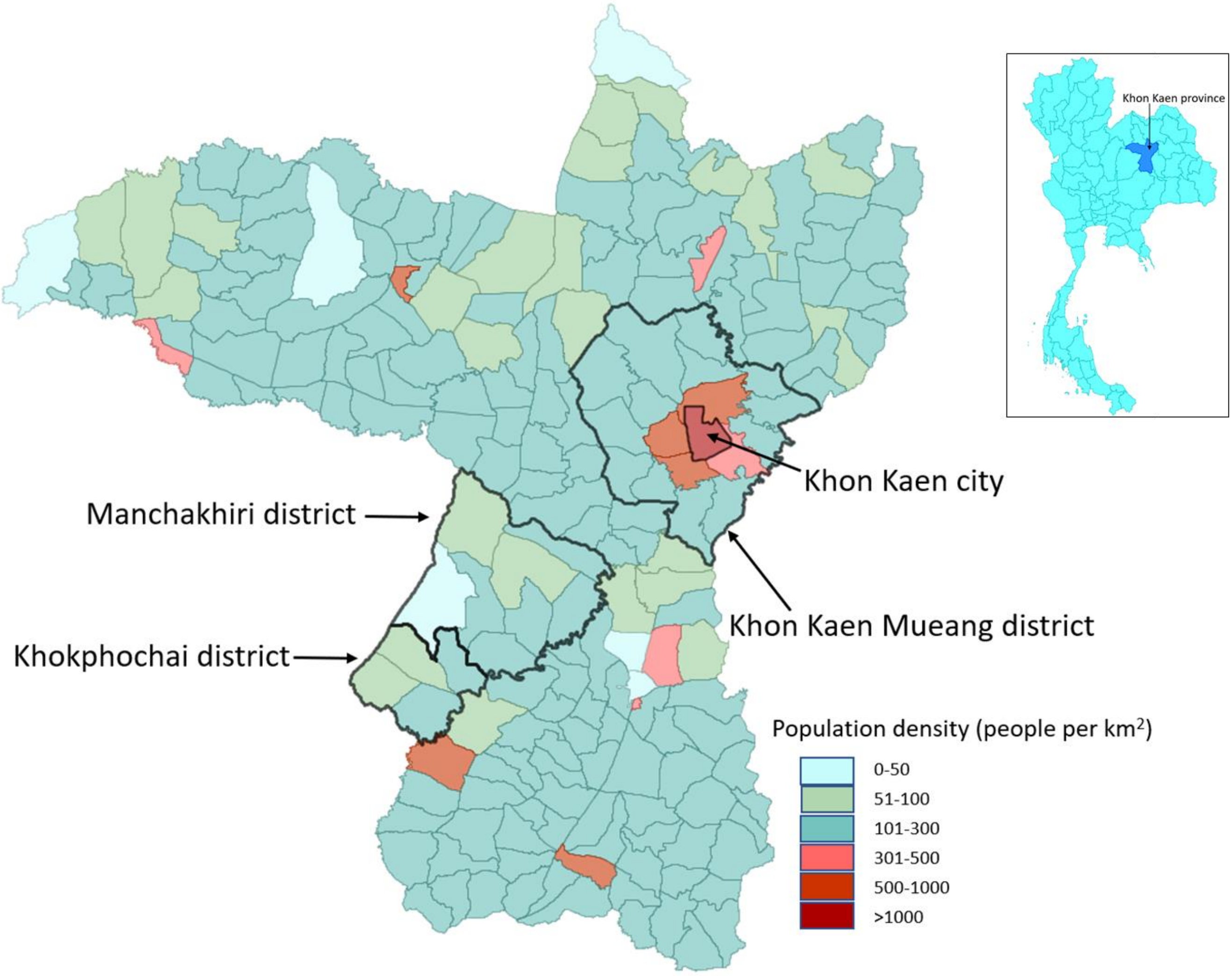

Additional file 1. Population density per sub-district, Khon Kaen province, Thailand, 2006 to 2016. Location of province in northeastern Thailand (inset).
