# Supplementary material for: Spatial and temporal patterns of dengue incidence in northeastern Thailand 2006–2016
Source: BMC Infect Dis. 2019 Aug 23;19:743. doi: 10.1186/s12879-019-4379-3 (PMC6708185; doi:10.1186/s12879-019-4379-3)
Supplement: Supplementary file 2 — Average monthly temperature (°C) per sub-district, Khon Kaen province, Thailand, January to December 2006–2016. (PDF 51 kb) [file 12879_2019_4379_MOESM2_ESM.pdf]

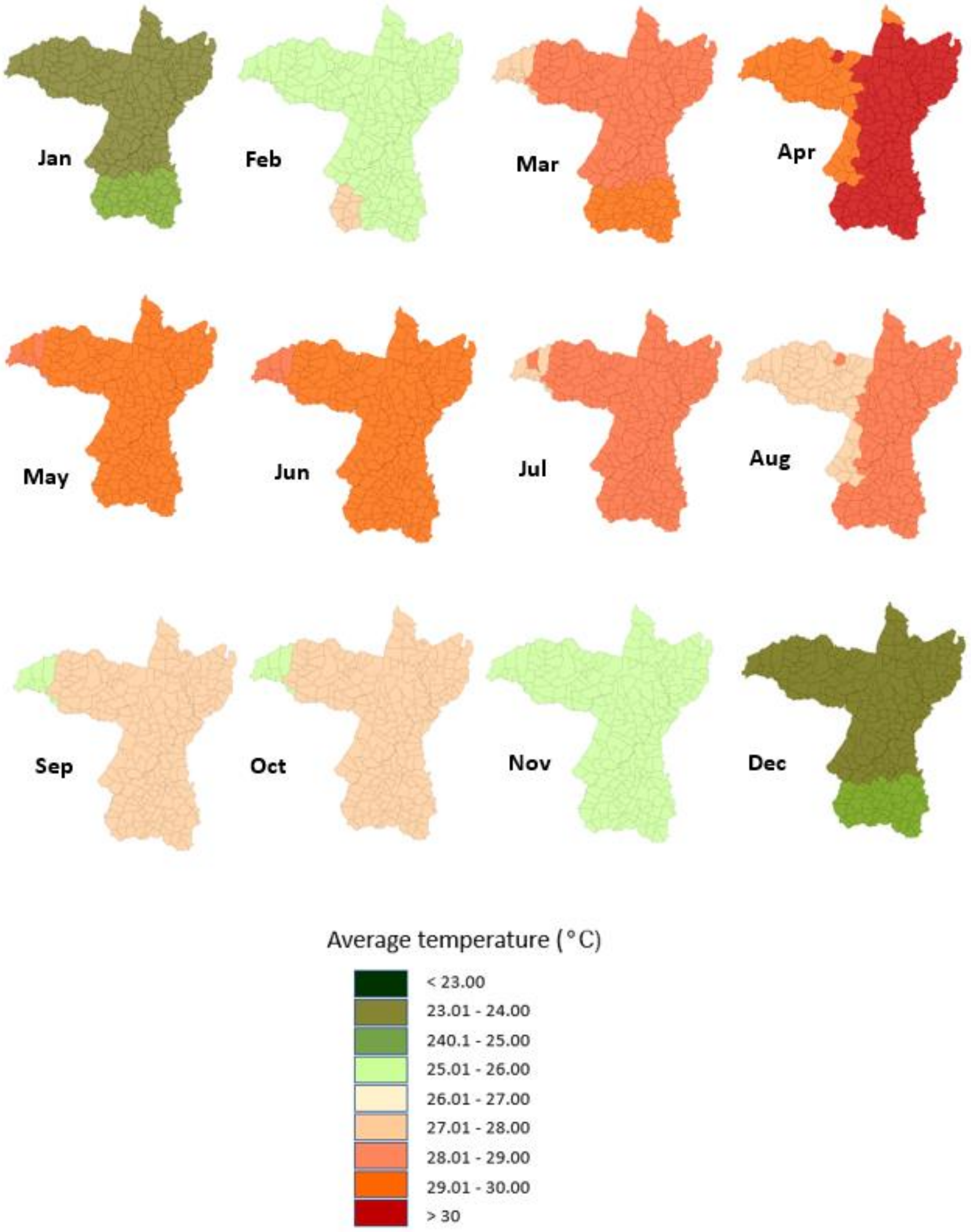

Additional file 2. Average monthly temperature (°C) per sub-district, Khon Kaen province, Thailand, January to December 2006-2016.
