# Supplementary material for: Spatial and temporal patterns of dengue incidence in northeastern Thailand 2006–2016
Source: BMC Infect Dis. 2019 Aug 23;19:743. doi: 10.1186/s12879-019-4379-3 (PMC6708185; doi:10.1186/s12879-019-4379-3)
Supplement: Supplementary file 3 — Average monthly rainfall (mm) per sub-district, Khon Kaen province, Thailand, January to December 2006–2016. (PDF 49 kb) [file 12879_2019_4379_MOESM3_ESM.pdf]

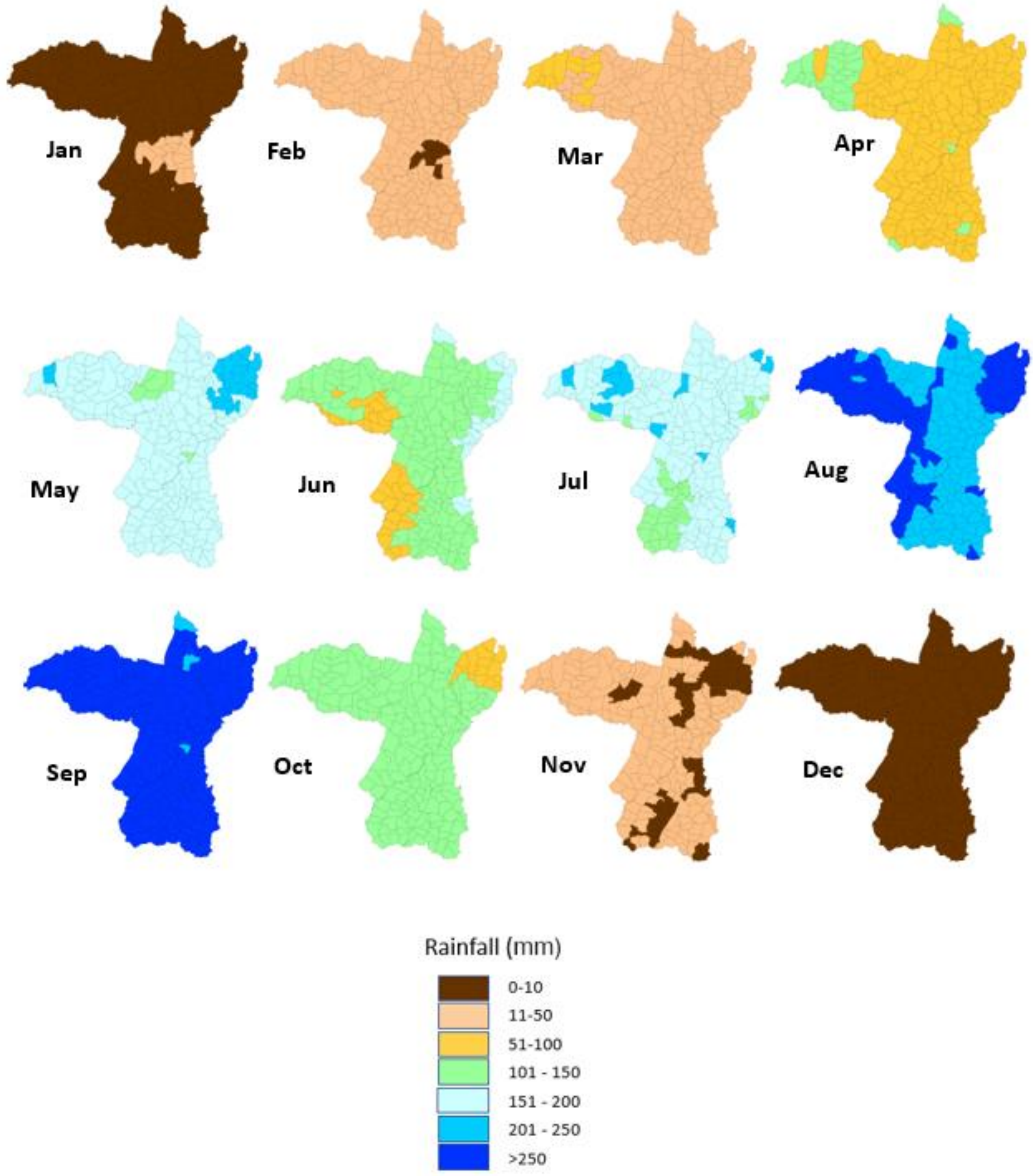

Additional file 3. Average monthly rainfall (mm) per sub-district, Khon Kaen province, Thailand, January to December 2006-2016.
