# Supplementary material for: Spatial and temporal patterns of dengue incidence in northeastern Thailand 2006–2016
Source: BMC Infect Dis. 2019 Aug 23;19:743. doi: 10.1186/s12879-019-4379-3 (PMC6708185; doi:10.1186/s12879-019-4379-3)
Supplement: Supplementary file 4 — Posterior distribution plots of A) Mean rainfall, B) Minimum temperature, C) Maximum temperature, D) Age, E) Gender, and F) Population density. (PDF 265 kb) [file 12879_2019_4379_MOESM4_ESM.pdf]

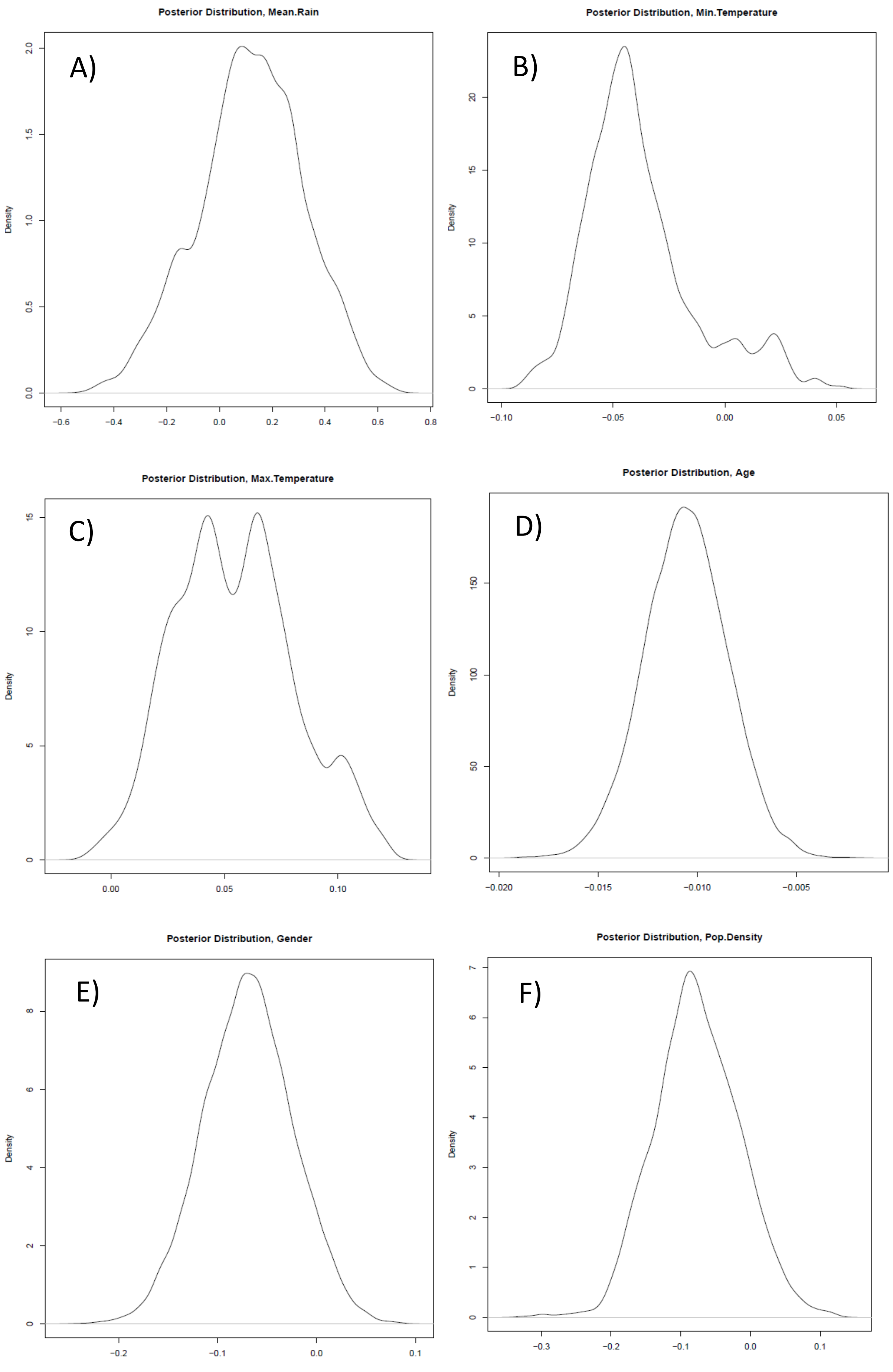

Additional file 4. Posterior distribution plots of A) Mean rainfall; B) Minimum temperature; C) Maximum temperature; D) Age; E) Gender; and F) Population density.
